# *Wolbachia* Prophage DNA Adenine Methyltransferase Genes in Different *Drosophila-Wolbachia* Associations

**DOI:** 10.1371/journal.pone.0019708

**Published:** 2011-05-06

**Authors:** Aggeliki Saridaki, Panagiotis Sapountzis, Harriet L. Harris, Philip D. Batista, Jennifer A. Biliske, Harris Pavlikaki, Stefan Oehler, Charalambos Savakis, Henk R. Braig, Kostas Bourtzis

**Affiliations:** 1 Department of Environmental and Natural Resources Management, University of Ioannina, Agrinio, Greece; 2 Department of Biological Sciences, University of Alberta, Edmonton, Canada; 3 Institute of Molecular Biology and Biotechnology, Foundation for Research and Technology-Hellas, Heraklion, Crete, Greece; 4 Technological Educational Institute of Kalamata, Kalamata, Greece; 5 Biomedical Sciences Research Center “Alexander Fleming”, Vari, Greece; 6 Medical School, University of Crete, Heraklion, Crete, Greece; 7 School of Biological Sciences, University of Bangor, Bangor, Gwynedd, United Kingdom; National Institutes of Health, United States of America

## Abstract

*Wolbachia* is an obligatory intracellular bacterium which often manipulates the reproduction of its insect and isopod hosts. In contrast, *Wolbachia* is an essential symbiont in filarial nematodes. Lately, *Wolbachia* has been implicated in genomic imprinting of host DNA through cytosine methylation. The importance of DNA methylation in cell fate and biology calls for in depth studing of putative methylation-related genes. We present a molecular and phylogenetic analysis of a putative DNA adenine methyltransferase encoded by a prophage in the *Wolbachia* genome. Two slightly different copies of the gene, *met1* and *met2*, exhibit a different distribution over various *Wolbachia* strains. The *met2* gene is present in the majority of strains, in *w*Au, however, it contains a frameshift caused by a 2 bp deletion. Phylogenetic analysis of the *met2* DNA sequences suggests a long association of the gene with the *Wolbachia* host strains. In addition, our analysis provides evidence for previously unnoticed multiple infections, the detection of which is critical for the molecular elucidation of modification and/or rescue mechanism of cytoplasmic incompatibility.

## Introduction

*Wolbachia pipientis* is an obligate intracellular symbiont belonging to the α-proteobacteria. It is thought to be present in an estimated 66% of all insect species, including disease vectors of animals and plants [1]. It has also been found in terrestrial isopods, spiders, mites, springtails and nematodes. Some *Wolbachia* strains can modify host reproduction and distort sex ratio by inducing parthenogenesis, feminisation, male killing or cytoplasmic incompatibility (CI) (reviewed in [2]).

*Wolbachia* phage particles were first observed by Wright et al. [3]. Masui et al. [4] described prophage WO, a genetic element in *Wolbachia* strain *w*Tai, containing about 26 open reading frames (ORFs) in 25 kb. The phage genome includes ORFs coding for capsid proteins, baseplate assembly proteins, integrase genes, several ankyrin-like proteins, as well as a potential methyltransferase. Further studies resulted in the isolation and characterization of the bacteriophage WO from the *Wolbachia* strain *w*CauB [5]–[7]. The genome of the purified phage is linear double-stranded DNA of about 43 kb, containing 47 ORFs (*w*CauB2) and 45 kb and 46 ORFs (*w*CauB3) [7]. Sequence analysis indicated that this phage genome includes ORFs coding for a DNA packaging protein, capsid proteins, baseplate assembly proteins, tail structural proteins and for several putative toxin-like secretory proteins [6], [7].

The *Drosophila melanogaster w*Mel genome was released in 2004 [8]. Since then, three other *Wolbachia* genomes have become available [9]–[11] and a number of other sequencing projects are currently in progress. Two divergent prophage WO families, WO-A and WO-B have been identified. Family WO-B can be divided into three clades [4], [12]. Distribution surveys indicate that WO-B homologs occur in at least 89% of the two main lineages of *Wolbachia* that infect arthropods [12], [13]. Five WO-B-like prophage regions are present in the *w*Pip genome, with some genes identical or highly similar between prophage copies, while other genes are unique. It seems likely that extensive recombination, duplication and insertion events have occurred between copies [10], [14]. In the highly recombining *w*Ri genome, 4 prophage segments have been detected [11], while no prophage elements could be identified in the mutualistic *w*Bm strain of the filarial nematode *Brugia malayi* [9]. Insertion sequences (IS) are frequently found in WO genomes and are considered to be a major factor driving phage recombination [8], [10], [11].

Since prophage regions have been found only in *Wolbachia* strains having a parasitic relationship with their hosts, it has been hypothesized that they contribute to the CI phenotype [4]. However, a study in different *Culex pipiens* strains detected no correlation between prophage *orf7* gene type and CI [15]. Another survey showed that phage associated protein Gp15 is similar to a bacterial virulence factor. This gene was partially correlated with CI expression, suggesting that it could be linked to a CI gene [16]. However, sequence analyses found no phylogenetic clustering of phage genotypes congruent with the four major *Wolbachia*-induced sexual alterations [13]. In *Nasonia vitripennis*, 12% of *Wolbachia* cells were found to show lytic phage development. The density of the bacteriophage correlated inversely with the density of *Wolbachia* bacteria [17]. While density is one of the most critical determinants of penetrance of *Wolbachia*-induced phenotypes [18]–[20], only virion-free *Wolbachia* were observed to contact the host spermatids [17]. These observations led to the Phage Density Model hypothesis, which suggests that lytic phages negatively control *Wolbachia* densities and expression of symbiont functions (reviewed in [21]).

Both WO-A and WO-B prophages in *w*Mel carry a gene that encodes a putative DNA adenine methyltransferase. Methyltransferase genes are carried by many bacteriophages [22]–[24], and modified bases are common in phage genomes [25], [26]. It is thus quite possible that a WO prophage methyltransferase has a phage-specific function or acts on the bacterial genome. Virulence and lysogeny of many pathogenic bacteria such as *Escherichia*, *Salmonella*, *Yersinia*, *Vibrio*, *Haemophilus*, *Pasteurella*, *Aeromonas*, *Actinobacillus*, *Klebsiella*, *Brucella* and *Rickettsia* are subject to control by adenine methylation [23], [27]–[29]. In some strains, methylation is essential for viability of the pathogen [30], [31]. Adenine methylation is also involved in cell-cycle regulation of bacteria [32]. In all these cases, methylation is performed by solitary or “orphan” methyltransferases, i.e. those that are not part of a restriction-modification (R-M) system. R-M systems protect bacteria against foreign DNA or act as selfish elements [33]. Negri et al. [34] provided the first evidence that a feminizing strain of *Wolbachia* interferes with host genetic imprinting through cytosine methylation of leafhopper DNA.

In the context of the fact that *Wolbachia* modifies the genome of its host insects, the presence of a DNA methyltransferase gene in its genome becomes even more intriguing. We therefore used molecular and phylogenetic approaches to detect and characterize the *Wolbachia*-phage embedded gene which encodes a putative DNA methyltransferase in a number of different *Wolbachia* strains of known CI properties. The ability of these strains to induce (*mod* status) and rescue CI (*resc* status) has previously been characterized [18], [35]–[38]. The potential involvement of this methyltransferase-like protein in host-*Wolbachia* symbiosis and in the induction of reproductive alterations is discussed.

## Results

### Sequence and distribution of the phage methyltransferase ORFs in different Wolbachia strains

**Figure 1:**
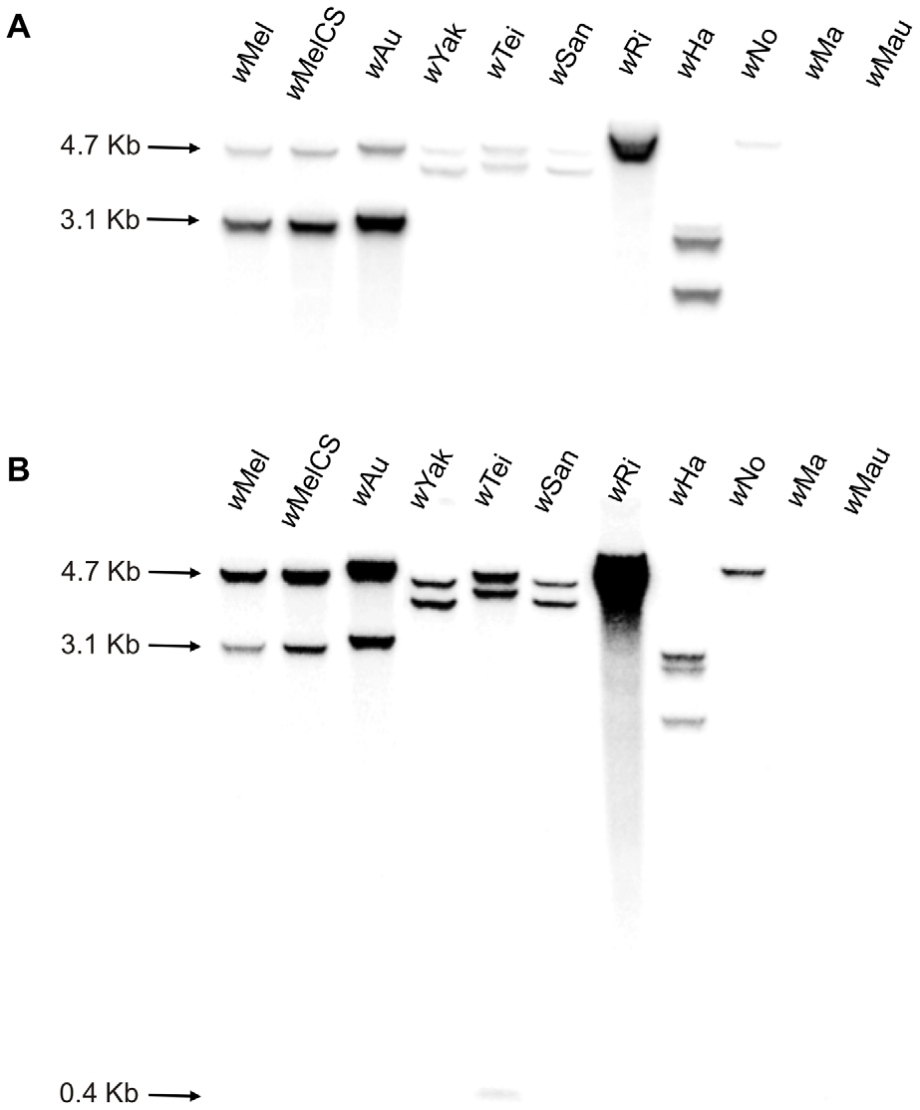
Southern blot analysis of *met* genes using genomic DNA from different *Wolbachia* infected species. The blot was hybridized with a probe corresponding to the full length *met1* (**A**) and *met2* (**B**) genes from *w*Mel.

Different *Wolbachia* strains of known CI phenotype (Table 1) were screened for the presence of the WO methyltransferase-like genes in order to test for a possible correlation with the CI phenotype. Degenerate and specific primers were designed for each of the two *w*Mel methyltransferase genes (Table 2) and used to amplify DNA from a number of different *Wolbachia*-infected *Drosophila* strains. *Wolbachia* strains *w*Mel, *w*MelCS, *w*Au and *w*Ha contain both methyltransferase ORFs. In contrast, strains *w*Ri, *w*No, *w*Yak, *w*Tei and *w*San only have one of the two ORFs (*met2*), while *w*Ma and *w*Mau do not contain any of the two methyltransferase ORFs. This was confirmed by Southern blot analysis using the two *w*Mel methyltransferase ORFs as probes (Fig. 1). The *w*Ha *met* ORFs were not further characterized.

**Table 1:** Insect lines and *Wolbachia* strains used in the present study.

Host species	Host strain	*Wolbachia* strain	Reference
*D. mauritiana*	Bloomington # 31	*w*Mau	[72]
*D. melanogaster*	yw^67C23^	*w*Mel	[18], [62]
*D. melanogaster*	Canton-S	*w*MelCS	[73]
*D. melanogaster*	Popcorn 3221	*wMelPop*	[74]
*D. santomea*	STO9, Africa	*w*San	[37]
*D. simulans*	Coffs Harbour	*w*Au	[36]
*D. simulans*	Riverside	*w*Ri	[42]
*D. simulans*	Hawaii	*w*Ha	[35]
*D. simulans*	Noumea	*w*No	[35]
*D. simulans*	KY203	*w*Ma	[75]
*D. teissieri*	Bloomington # 1015	*w*Tei	[37]
*D. yakuba*	SA3, Africa	*w*Yak	[37]
*D. simulans*	STCP line 2	*w*Tei	[38]
*D. simulans*	STCP line 4	*w*Tei	[38]
*D. simulans*	STCP line 1	*w*San	[38]
*D. simulans*	STCP line 14	*w*Yak	[38]

**Table 2:** PCR primers used in the present study.

Primer	Sequence (5′→3′)	Tm
wMeth12_ext_F	CTTCTYYAGCGTCAGASRTWTTTT	54
wMeth12_ext_R	CTCTTGCCCATTCYGTYTGYGTGA	54
wMeth12_int_F	ATGAAYTTAGCAAYMCACTAC	54
wMeth12_int_R	CTTCTTGAATTTSKGCAAA	54
meth2_F2	CTTCTTTAGCGTCAGAGATA	56
meth2_R2	GCCCATTCCGTTTGTGTGAT	60
meth2_F1	TTCAGCCAGAATGGCGGATT	60
meth2_R1	ACATATTGATTTGAAACTCC	52
meth1-F	ACATAGCAATTTAATACACTA	52
meth1-R	GTCTGCGTGATTTTTCCTCC	60
met_102F	CAGGGAATTTGGCTTTCGTA	58
met_269R	GATTTGCCAGTAACCGAAAA	56
met_102F	CAGGGAATTTGGCTTTCGTA	58
met_1024R	CCCCAGGTCTGCTGCTATTA	62
TeiB_1024R	CTCCTGATCTGCTGCTGTTT	60

The *met1* ORFs are more closely related to each other than to the respective *met2* ORFs. ORF *met2_w*Au contains a 2 bp GC deletion after codon 92, leading to a truncated ORF lacking the C-teminal methyltransferase domain.

**Figure 2:**
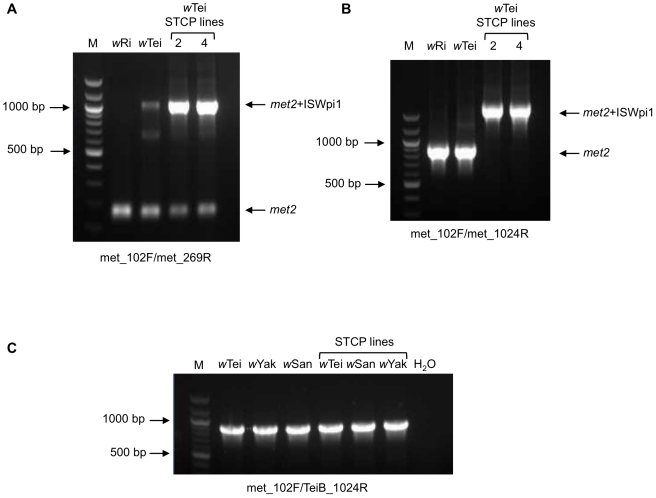
PCR analysis of *met2* copies in *Wolbachia* strain *w*Tei. PCR was performed using primers met_102F and met_269R (**A**), met_1024R (**B**) or TeiB_1024R (**C**). Primers met_102F and met_269R detect both A-group- and B-group-like *met2*, met_1024R detects only A-group-like *met2*, while TeiB_1024R detects only B-group-like *met2*. M: 100 base pair molecular weight DNA marker (New England Biolabs).

Further PCR analysis using met_102F/met_269R primers indicated the presence of three PCR products in *w*Tei: While the first was of the expected size for a *met2* product, the other two were about 500 and 900 bp larger (Fig. 2A). Sequencing of the largest product revealed the presence of ISWpi1, a *Wolbachia*-specific insertion sequence belonging to the IS5 family [39], in *met2* gene. The intermediate sized PCR product could not be cloned in several attempts, probably because of the presence of unstable repeat sequences. Surprisingly, when PCR with the same primers was performed using DNA from *D. simulans* STCP flies transinfected with *w*Tei [38], only two PCR products were detected, with a striking difference in the intensity ratio (Fig. 2A).

Cloning and sequencing of the methyltransferase ORFs from the closely related *w*San, *w*Tei and *w*Yak strains also detected two different *met2* ORFs; one closely related to the *met2* sequences of supergroup A *Wolbachia* strains (*met2_w*TeiA for *w*Tei), while the other is closely related to B supergroup strains (*met2_w*TeiB for *w*Tei). Specific reverse primers were designed to anneal only to the A-group-like *met2* (met_1024R; Fig. 2B) or the B-group-like *met2* (TeiB_1024R; Fig. 2C). PCR reactions with these primers revealed that strains *w*San, *w*Tei and *w*Yak bear a copy of the B-group-like *met2*; these genes were successfully transferred by microinjections into *D. simulans* STCP lines by Zabalou et al. [38] (Fig. 2C). The ISWpi1-disrupted *met2* ORF of *w*Tei was also transferred together with *w*Tei into STCP, but not the intact copy of *met2_w*TeiA (Fig. 2B).

### Phylogenetic Analysis

**Figure 3:**
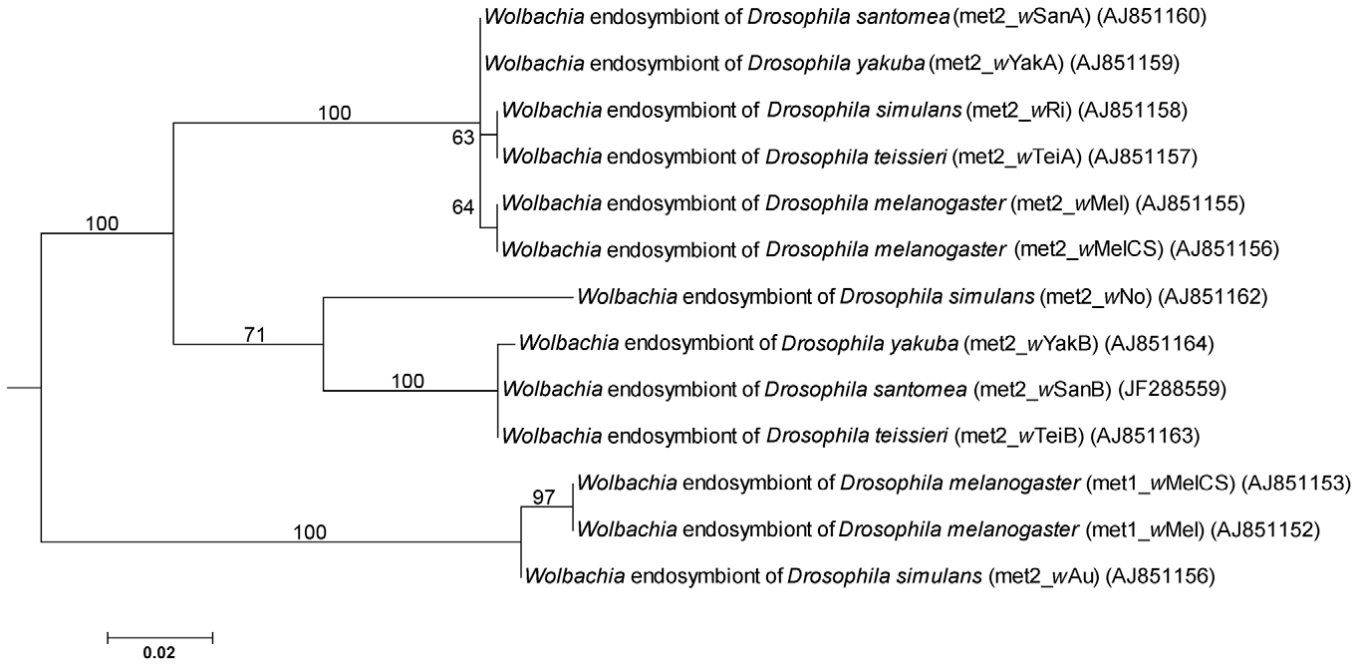
Phylogenetic tree of *Wolbachia* based on *met* gene sequences. The tree was constructed by Maximum Likelihood analysis. Numbers on the nodes indicate bootstrap values.

All methods used to reconstruct phylogenies yielded similar results. The three methods, distance, parsimony and maximum-likelihood (ML), make different evolutionary assumptions, thus their congruence provides strong support for the deduced phylogeny. We show only the tree derived by ML estimation (Fig. 3). The phylogenetic clustering of the *met2* ORFs is similar to the currently accepted clustering of the respective *Wolbachia* strains: all *met2* gene sequences coming from supergroup A strains cluster together (*w*Mel, *w*MelCS, *w*Ri, *w*Au, *w*Tei, *w*Yak and *w*San strains). The *met2* gene sequences from supergroup B strains also form a cluster and are distantly related to the supergroup A gene sequences. This suggests a long association of the methyltransferase genes, and consequently of the phages harbouring them, with the respective *Wolbachia* strains.

### RT-PCR analysis

**Figure 4:**
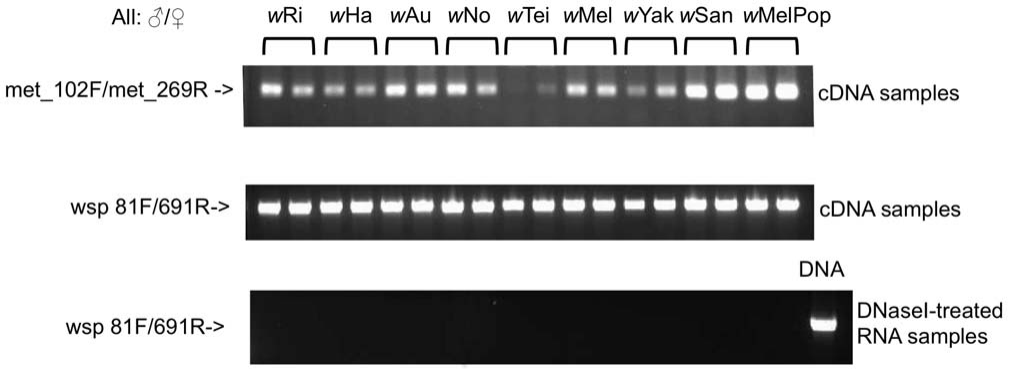
RT-PCR analysis of *met2* genes. RNA samples were prepared from young male/female adult flies from different *Drosophila/Wolbachia* associations. The bottom panel presents the control samples for the presence of genomic contamination.

Transcriptional analysis of *met2* genes was performed by RT-PCR on cDNA samples prepared from young adult male and female flies. *Met2* transcripts were detected in all samples tested (Fig. 4). The *met2* copy of *w*Tei, which is disrupted by ISWpi1, was found to be transcriptionally silent (data not shown).

## Discussion

The release of the first *Wolbachia* genome (*w*Mel strain) revealed that it contains two DNA methyltransferase genes *met1* and *met2*, encoded by two prophages, WO-A and WO-B respectively [8]. This finding is intriguing in the light of the fact that *Wolbachia*-induced CI involves modification of the insect host chromosome [40]. The presence of phage-like particles in *Wolbachia*-infected hosts [3], [5], [17], [41] suggests an active role of the phage in *Wolbachia* biology. Thus, it is tempting to speculate that, beyond controlling lysogeny of the phage, the methyltransferases might be involved in triggering reproductive alterations imposed by *Wolbachia* on its host. We therefore undertook a survey of *Wolbachia* strains of known CI status for the presence of the methyltransferases, determined their sequences and reconstructed their phylogeny.

The *met1* gene is only present in a few of the tested strains; there is no correlation with CI. *Wolbachia* strains *w*Mel, *w*Ri, *w*No and *w*Pip induce CI in permissive hosts [35], [42]–[44], and they all contain at least one functional copy of the *met2* gene. In contrast, strain *w*Au [36] does not induce or rescue CI and its only *met2* ORF is disrupted. *Wolbachia* strains *w*Mau and *w*Ma do not contain the *met2* gene and also do not induce CI in their hosts [43], [45], [46]. On the other hand, *w*Yak, *w*Tei and *w*San have recently been shown to fully rescue the *w*Ri modification, while they are unable to induce CI in their native hosts [37]. *w*Tei is, however, able to induce 100% CI after transfer into the permissive host *D. simulans* [38]. In this context, it is interesting to note that our data suggest a double or multiple infection of the original host of *w*Tei, of which not all *Wolbachia* strain(s) were transferred upon transinfection of *D. simulans* (Fig. 2). Cloning and sequencing of PCR products, using different methyltransferase primer sets, supports the presence of a hidden double infection of *D. teissieri*. This could explain the phenotypic shift in CI properties that is observed between the natural host and the engineered strains.

When the rescue properties of all A group strains are examined, *resc^−^* strains lack a functional A-group-like *met2* gene, which is always present in *resc^+^* strains (Table 3); a possible correlation between *met2* and CI rescue should therefore be considered. B group strains (*w*No, *w*Ma, *w*Mau) are all *resc^+^*, nevertheless they do not possess an A-group-like *met2* gene. The genomes of these strains have not been sequenced and the presence or absence of prophage copies has not yet been documented. While *w*Ma and *w*Mau do not contain any *met* gene, *w*No has a B-group-like *met2* ORF; this could reflect a different mechanism regulating CI in B group *Wolbachia* strains.

**Table 3:** Distribution of phage methyltransferases in *resc*+ and *resc*− *Wolbachia* strains.

*Wolbachia* strain	CI rescue	*met1*	A group-like *met2*	B group-like *met2*	Disrupted *met2*
*w*Mel	+	+	+	−	−
*w*MelCS	+	+	+	−	−
*w*Tei	+	−	+	+	by ISWpi1
*w*Yak	+	−	+	+	−
*w*San	+	−	+	+	−
*w*Ri	+	−	+	−	−
*w*Au	−	+	−	−	stop codon
*w*Tei (STCP)	−	−	−	+	by ISWpi1

Transgenic expression of *w*Mel *met2* (WD0594) in *D. melanogaster* was recently reported using the UAS/GAL4 system [47]. This study revealed no modification of phenotype in flies expressing *met2* ubiquitously and, similarly, when expressed specifically in the ovaries, no rescue phenotype was apparent in CI crosses. Although these data suggest that constitutive expression of the *met2* gene does not alone drive the CI phenotype, it is still unclear what type of regulation *met2* or any of the phage-related genes are subject to and how this affects the mechanism of CI.

Southern blot analysis indicates the presence of a *met*-like gene also in the *Wolbachia* strain *w*Uni, which is known to induce parthenogenesis in the parasitic wasp *Muscidifurax uniraptor* (data not shown). The distribution of the *met* gene in parthenogenesis-inducing *Wolbachia* strains remains to be investigated. Interestingly, the mutualistic *Wolbachia* strain, which is present in the filarial nematode *Brugia* malayi, neither induces reproductive alterations nor carries a copy of the DNA methyltransferase genes.

Additionally, and important for any interpretation of the role of *met2*, we demonstrated expression of the gene in all *Wolbachia* strains with RT-PCR (Fig. 4). The *Wolbachia* phage DNA methyltransferase may be involved in the methylation of phage, bacterial, insect host genes or a combination of them. Although *Drosophila* had for a long time been considered to be free of DNA methylation, both the presence of methyltransferase genes in its genome [48], [49], and of 5-methylcytosine residues in the early stages of embryonic development [50], [51] have been demonstrated. Interestingly, a Dam-like methyltransferase has been implicated in male sterility in plants [52].

Base modification in bacterial genomes is performed by two classes of DNA methyltransferases: (i) those associated with restriction-modification systems, and (ii) solitary methyltransferases that do not have a restriction enzyme counterpart. Examples of the latter are the N6-adenine methyltransferases Dam and CcrM [53], [54]. In α-Proteobacteria, CcrM methylation regulates the cell cycle in *Caulobacter crescentus*, *Rhizobium meliloti* and *Agrobacterium tumefaciens* and plays a role in *Brucella abortus* infection (reviewed in [55]). Overexpression of CcrM in these bacteria results in the accumulation of multiple chromosomes, indicative of overinitiation of DNA replication [56], [57]. *Wolbachia* prophage methyltransferase could regulate several aspects of the symbiont's cell cycle by imposing a specific epigenetic signal.

*In silico* analysis of *Wolbachia* prophage methyltransferase has predicted an N-terminal ParB-like nuclease domain (data not shown) similar to the ParB of the *parCBA* operon in *E. coli*, which is important for plasmid stability and resolving dimeric or multimeric plasmids. ParB nucleases have also been reported in several other plasmid genomes. ParB nucleases are Ca++ dependent endonucleases with 5′ -3′ exonuclease activity [58], [59].

**Figure 5:**
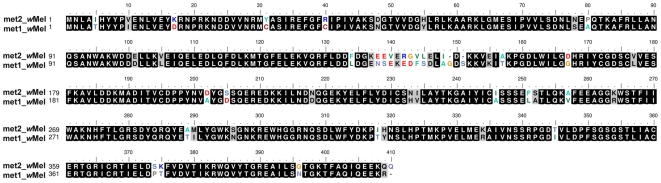
Amino acid alignment of Met1 and Met2 proteins of *Wolbachia* strain *w*Mel. Black highlight indicates amino acid identity; grey highlight indicates amino acid similarity.

The methyltransferase genes, *met1* and *met2*, are closely related (Fig. 5). The phylogenetic clustering of the methyltransferase genes, in particular of the *met2* gene, is similar to the currently accepted clustering of the arthropod *Wolbachia* strains, both on the level of the major division of the *Wolbachia* strains into two supergroups, A and B, as well as on the lower level of clades and strains. Specifically, the *met2*-based tree is similar to the respective *wsp*-based tree (data not shown). This suggests a long association of the methyltransferases, and consequently of the phages carrying them, with the harbouring *Wolbachia* chromosomes (Fig. 3). However, translocation of *met2* gene from the phage genome to *Wolbachia* chromosome cannot be excluded for any of the strains studied; such an event could explain why *met2* phylogeny correlates with *Wolbachia* phylogeny.

*Wolbachia* exhibits a fascinating array of host manipulations. The elucidation of the molecular basis of the host-symbiont interaction will allow insight in the regulation of fundamental cell biological processes. Future studies will address any potential direct or indirect effect of the methyltransferase(s) in the establishment of symbiosis and/or the induction of reproductive manipulations.

## Materials and Methods

### Insect lines

Insect lines and *Wolbachia* strains used in the present study are listed in Table 1. Flies were routinely grown at 25°C on standard cornmeal medium in uncrowded vials. The *Drosophila simulans* STCP lines were produced by Zabalou et al. [38] who transferred *Wolbachia w*Yak, *w*Tei, *w*San strains into the same host background using embryonic cytoplasmic injections.

### PCR analysis

DNA was extracted from adult flies using the NucleoSpin Tissue kit (Macherey-Nagel) or the CTAB protocol, as previously described [60]. The DNA was used as template for PCR reactions and Southern blot analysis. About 50 to 100 ng total DNA from adult flies were used as template in PCR reactions of *Wolbachia* targets. The presence of *Wolbachia* was initially determined using *Wolbachia*-specific 16S rDNA primers [61]. The primers used to amplify methyltransferase gene sequences are listed in Table 2. Standard PCR analysis was performed using GoTaq® Flexi DNA polymerase (Promega). To generate DNA templates for sequencing, PCR reactions were done using the Elongase Amplification System (Invitrogen, Glasgow, UK). The PCR products were A-tailed, cloned into the pGEM-T Easy vector (Promega, Wisconsin, USA) and transformed into competent *E. coli* XL1-Blue MRF cells (Stratagene, Amsterdam, The Netherlands). Plasmid DNA was extracted using the Nucleospin Plasmid kit (Macherey-Nagel). Sequencing reactions were performed with the SequiThermTM Excel Long ReadTM DNA sequencing Kit-LC (Epicentre Technologies) and the DNA sequence of the inserts was determined at the laboratory of Microchemistry, FoRTH, Heraklion (Greece) on a LiCor 4200 DNA sequencer. Three to six clones were sequenced from each individual. The methyltransferase gene sequences of this study have been deposited in the EMBL database under the accession numbers AJ851152 to AJ851164 and FR796473, and in the GenBank database under the accession number JF288559.

### Southern blot analysis

Genomic DNA was prepared as reported previously [62] and digested with *Hae*III. Agarose gel electrophoresis of DNA and blotting to nylon membranes were carried out using standard procedures [63]. DNA probes were prepared by random hexanucleotide priming [64]. Hybridization of ^32^P-labelled probes to blotted DNA was performed using standard procedures [63].

### RT-PCR analysis

For each of the tested *Wolbachia-Drosophila* associations, total RNA from young male and female adult flies was extracted using TRIzol (Invitrogen) and treated with RNase-free DNase (Invitrogen). First-strand cDNA was synthesized from 5 µg of total RNA using reverse transcriptase (SuperScript III; Invitrogen) and random primers (Promega) and the reactions were treated with RNase H. RNA integrity was assessed using the universal *Wolbachia wsp* 81F/691R primers [65]. All RNA samples were tested for genomic DNA contamination by performing PCR using *wsp* 81F/691R primers in DNaseI-treated RNA samples which were not reverse transcribed. Transcription of *met2* was detected using GoTaq® Flexi DNA polymerase (Promega).

### Alignment and Model Selection

*Wolbachia* adenine methyltransferase nucleotide and amino acid sequences were aligned using the ClustalW Multiple Alignment algorithm implemented in Geneious v.5.3.3. [66]. The appropriate evolutionary model JTT+ Γ was selected by the Akaike Information Criterion (AIC) using ProtTest v.2.4 [67]. Models of substitution for nucleotide alignments were selected using AIC in jModeltest v.0.1.1. [68]. The appropriate evolutionary model was TPM1uf+I+Γ.

### Phylogenetic analysis

The evolutionary history was inferred by maximum likelihood criterion using PAUP* v.4.0b10 for the nucleotide alignment and PHYML for the protein alignment [69], [70]. Phylogenetic trees were generated using ML bootstrap analysis in PAUP (100 pseudoreplicates of heuristic search with 10 random sequence). The maximum likelihood method conducted using PHYML was performed using 100 bootstrap replicates, a fixed proportion of invariable sites, an estimated gamma distribution parameter and optimized topology, branch lengths and rate parameters. ML trees generated are midpoint rooted using Archaeopteryx v. 0.957b [71].

### Methyltransferase nomenclature

The methyltransferase gene sequences were named based on the following system. Each methyltransferase gene name is composed of “*met*” (in italics) denoting methyltransferase, followed by the numbers 1 or 2, indicating their origin from phage WO-A or WO-B, respectively (phage nomenclature according to [8]) and concluded by the name of the *Wolbachia* strain harbouring the methyltransferase gene (eg *w*Ri, *w*Mel, etc). Using this nomenclature, the two methyltransferase genes present in the *w*Mel strain are named *met1_w*Mel and *met2_w*Mel.
